# Drilling Capability of Orthodontic Miniscrews: In Vitro Study

**DOI:** 10.3390/dj8040138

**Published:** 2020-12-21

**Authors:** Alessandra Marchi, Matteo Camporesi, Maurizio Festa, Luis Salvatierra, Sara Izadi, Giampietro Farronato

**Affiliations:** Department of Orthodontics, Fondazione IRCCS Cà Granda, University of Milan, 20122 Milan, Italy; matteo.camporesi@unifi.it (M.C.); maurizio.festa@unimi.it (M.F.); Luissalvatierra@outlook.it (L.S.); sara.izadi@unimi.it (S.I.); giampietro.farronato@unimi.it (G.F.)

**Keywords:** orthodontic, miniscrew, in vitro study, torque

## Abstract

The aims of this study were to assess the values and mechanical properties of insertion torque (IT) of steel miniscrews inserted in artificial bone blocks (Sawbones, Pacific Research Laboratories, Vashon, WA, USA) with different bone densities and to detect any scratches on the surface of the miniscrews after insertion. Forty self-drilling miniscrews (Leone S.p.A. ø 1.75 mm, L 8 mm) have been inserted into bone blocks that mimic different stability conditions (density: 20 PCF—pounds per cubic foot, 40 PCF, and 30 + 50 PCF with 2 mm and 4 mm of cortical bone). Before insertion and after removal, all miniscrews were inspected with a stereomicroscope 5x and a SEM to detect potential microscopic cracks. Using an electronic surgical motor (W&H Dentalwerk Bürmoos GmbH, Werner Bader Str. 1, 5111 Bürmoos, Austria), the maximum insertion torque value was registered. Stereomicroscope and SEM examination did not indicate any morphological and surface structural changes to the miniscrews, irrespective of the bone density they were inserted into. The findings showed that IT increased significantly with increasing bone density. In each artificial bone block, morphostructural analysis demonstrated the adequate mechanical properties of the self-drilling miniscrews. IT measurements indicated torque values between 6 and 10 Ncm for blocks with a density of 30 + 50 PCF, whereas the suggested values are between 5 and 10 Ncm.

## 1. Introduction

Anchorage, an integral factor for limiting orthodontic movement to only some dental components, is one of the most critical aspects of orthodontic treatment, taking advantage of the stable resistance of other elements [1,2,3].

Orthodontic miniscrews (also referred to as microscrews, micro/mini-implants, orthodontic implants, or TADs—temporary anchoring devices) are devices specifically designed to be mounted within the bones in order to provide orthodontic device anchorage [4,5,6]. Orthodontic skeletal anchorage study has contributed to the development of intraoral devices capable of achieving absolute anchorage: miniscrews, also known as TADs (temporary anchorage devices). Different models of mini orthodontic screws have been developed over the years with the goal of enhancing biomechanical features and increasing their clinical efficacy. More recently, simple-to-use surgical miniscrews (lightweight, self-drilling or self-threading machines) have been developed that are easy to use, highly biocompatible, and resistant to loads and corrosion. The peculiarity of these devices lies in the ability to establish, once implanted, mechanical retention (primary stability) without specifying bone osteointegration, which makes it simpler and less invasive to remove the device at the end of therapy. Insertion torque is associated with primary stability during miniscrew placement [7], but excessive torque can lead to cracks in the cortical bone and to bone reabsorption, resulting in miniscrew failure [8,9]. Many authors refer to maximum insertion torque (MIT) values ranging from 5 to 10 N/cm, the maximum value of the insertion torque measured during the miniscrew insertion [5,7,8,9].

The initial stability of a miniscrew is important because the majority of orthodontic miniscrew failure incidences occur in the early stages [10]. The shift of a screw can occur due to inflammation as a result of a weak bone–screw interface, but screw factors are also considered critical for their initial stability, such as the diameter of the screw, weight, diameter of the pilot hole, and shape of the screw thread [11,12,13].

A calculation of the insertion torque is important to predict the initial stability of a miniscrew. Insertion torque is widely used to determine the mechanical stability of implants, including miniscrews [14,15,16,17,18,19].

Previous studies have shown that in order to achieve initial anchorage at the screw and bone interface, a certain degree of insertion torque is required, and the insertion torque of miniscrews is a significant factor in determining the appropriate initial stability of a screw [17,20]. In addition, excessive insertion torque, heat at the border between the screw and the bone, and mechanical damage have been suggested to cause bone degeneration at the implant–tissue interface [21]. Nguyen et al. stated that the ideal penetration torque range of 5–10 Ncm endorsed in the literature seems to be feasible only when the thickness of the cortical bone is between 0.5 and 1.0 mm [22,23].

The purpose of this research was to test the biomechanical characteristics of self-drilling steel miniscrews, specifically, to study the values of insertion torque at different densities of synthetic bone, the instrumentation historically used in prosthetic implantology. We want to prove that self-drilling miniscrews can be inserted into high-density bone without excessive development of heat, despite being inserted in bone without a pre-drilled hole. The insertion of miniscrews in the high-density maxillary zone without the need to first make a hole is of clinical importance. Additionally, a scanning electron microscope and a 5x stereomicroscope were used to investigate possible structural changes in the screws after the stress. By means of this study, the drilling capability of self-drilling miniscrews was evaluated. This study was conducted to test the hypothesis that the self-drilling miniscrews do not have the mechanical properties to enable drilling of the cortical bone without also generating excessive torque and bone damage.

## 2. Materials and Methods

**Figure 1:**
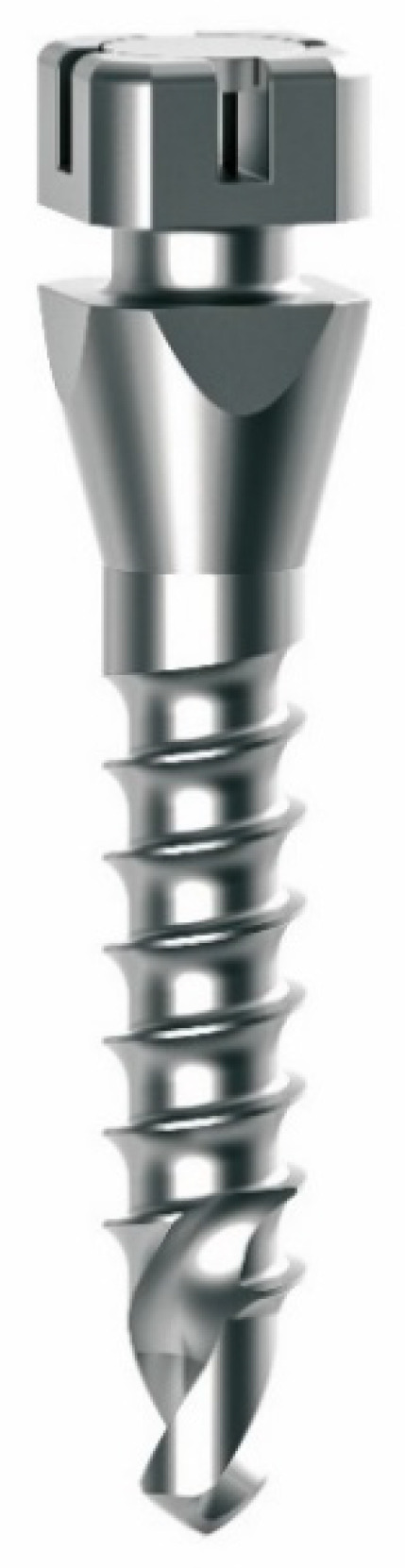
Self-drilling miniscrews used in the study (diameter of 1.75 mm, length of 8 mm, and high head).

Forty stainless steel self-drilling orthodontic miniscrews (Leone SpA, Sesto Fiorentino, Florence, Italy) were tested. All miniscrews had a diameter of 1.75 mm, a length of 8 mm, and a high head (Figure 1).

Synthetic bony blocks made of rigid polyurethane foam (Sawbones, Pacific Research Laboratories Inc., Vashon, WA, USA) were used in order to simulate different qualities of bone.

Four different bone block types were used:

- Sample 1. Blocks composed of a cortical layer with a density of 0.64 g/cm^3^ (40 pounds per cubic foot (PCF)) and a thickness of 2 mm, and a cancellous bone layer with a density of 0.32 g/cm^3^ (20 PCF);
- Sample 2. Blocks composed of a cortical layer with a density of 0.64 g/cm^3^ (40 PCF) and a thickness of 4 mm, and a cancellous bone layer with a density of 0.32 g/cm^3^ (20 PCF);
- Sample 3. Blocks composed of a cancellous bone layer with a density of 0.32 g/cm^3^ (20 PCF);
- Sample 4. Blocks composed of a cortical bone layer with a density of 0.64 g/cm^3^ (40 PCF).

The selection of the thickness values of the cortical layer was based on mean thickness studies for the maxilla and mandibula. The mean maxilla cortical thickness was 1.49 +/− 0.34 mm (n = 98, range 0.92–2.54), and the mean maxilla cortical thickness was 2.22 +/− 0.47 mm (n = 127, range 0.79–3.21), recorded by Miyamoto et al. The cortical layer thicknesses chosen for this analysis were 2 and 4 mm based on these parameters [24].

**Figure 2:**
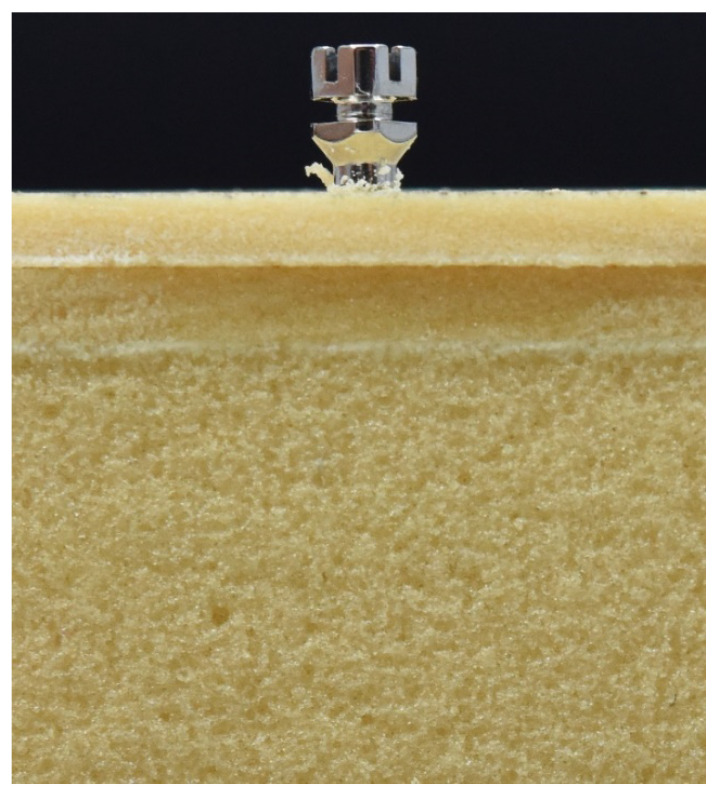
The bone block and the inserted miniscrew.

The miniscrews were positioned into the synthetic bony block to measure maximum insertion torque (MIT) with a 30:1 contra-angle mounted over a surgical handpiece (Implantmed^®^, W&H Oral Surgery, Bérmoos, Austria) at a rotating speed of 15 rpm. Care was taken to position the tilted screw at an angle of 90° with respect to the block surface and insert the miniscrew until the end of the threaded portion (Figure 2).

To guarantee homogeneity and perpendicularity of insertion, a specific device was created. It allows axial coincidence among screw and sintetic bone to be obtained [25].

Before each measurement, we calibrated torque gauge. All miniscrews, before being inserted, were photographed with stereomicroscope Nikon SMZ800 (Nikon Instruments Inc., Melville, NY, USA) and SEM Quanta 200 (FEI Quanta 200, Eindhoven, The Netherlands), provided by Leone S.p.A.

Finally, after being removed from the substrate, the miniscrews were re-evaluated and photographed with stereomicroscope and SEM to detect any surface structural alterations to the miniscrews caused by the insertion and removal of them from the bone specimen.

## 3. Statistical Analysis

To measure the minimum sample size for each group, the fixed effects ANOVA one-way statistical test was used. This estimate was based on previous research results, taking into account a significance level of 0.05 and a test intensity of 80% [26]. The minimum size of each group was ten miniscrews. The Student’s *t*-test was used to estimate discrepancies in variables between the four classes and the 10 Ncm reference value. The significance level was set at *p* < 0.05.

## 4. Results

**Figure 3:**
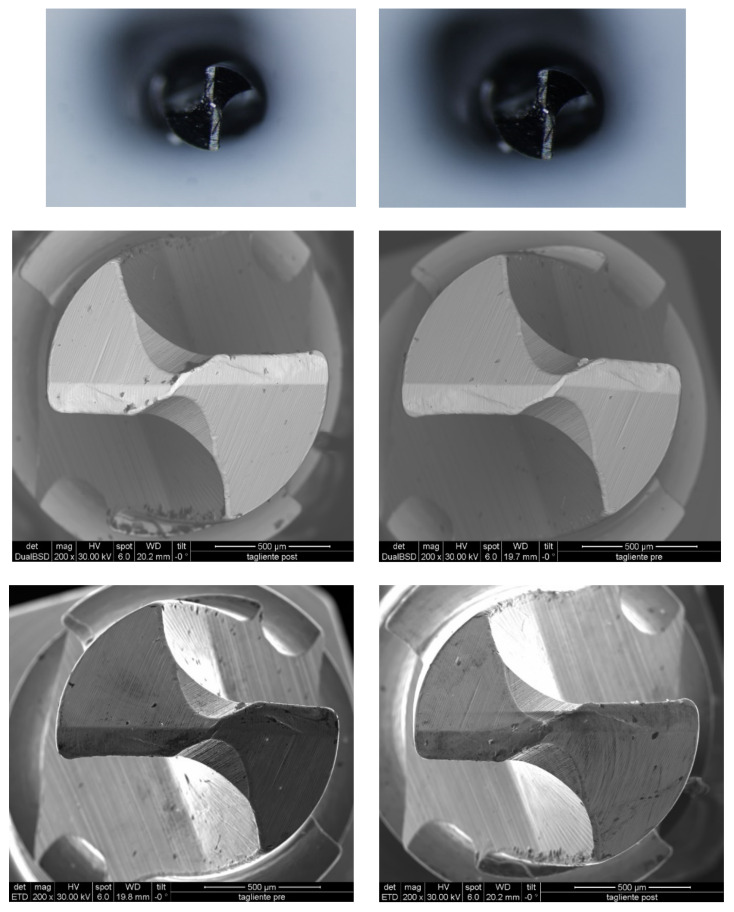
Images of miniscrews by stereomicroscope and SEM before and after insertion in the artificial bone block composed of a cortical bone layer with a density of 0.64 g/cm^3^ (40 PCF). The authors performed a qualitative evaluation by examining only the behavior of the miniscrew in the bone block with greater density (0.64 g/cm^3^, 40 PCF). This is the most stressful test for miniscrews. If the screw does not undergo deformation during this test, it will not undergo any deformation in the lower-density blocks. Mean values for maximum insertion torque (MIT) showed different MITs for the different insertion blocks. With all the screws, the MIT increased with increasing cortical bone thickness (Figure 4).

The morpho-structural analyses detected no alterations of the miniscrews’ surface with SEM and stereomicroscope, with the same result for all samples (Figure 3).

In samples with 20 PCF density, maximum torque values were reached below 1 mm deep and had a linear pattern, without dropping below 2 Ncm +/− 0.8.

In samples with 40 PCF density, the torque had an incremental trend throughout the insertion thickness. The maximum torque value was above 19 Ncm (mean: 19.3 Ncm +/− 1.5).

In samples with 30 PCF density and 2 mm cortical 50 PCF, torque values had an incremental trend until 2 mm of insertion, with values increasing up to 6 Ncm (6.8 Ncm +/− 0.5), which remained constant until fully inserted.

In samples with 30 PCF density and 4 mm cortical 50 PCF, torque values had an incremental performance until 3.5 mm of insertion and retained the value of 10 Ncm until fully inserted (10.0 Ncm +/− 0.8) (Table 1).

## 5. Discussion

Presented by Heidemann in 1998 [27], self-drilling is designed to allow screws to be directly inserted into the bone substrate through the mucosa, by means of a manual screwdriver or surgical micromotor. For these miniscrews, the creation of the pilot hole is often unnecessary. They are characterized by greater primary stability and a better BIC (bone implant contact); their insertion causes the creation of numerous bone fragments between the coils that can be interpreted as evidence that the bone is not compressed, but etched [28]. The miniscrews analyzed do not require predrilling, so they are under greater mechanical stress during the insertion [22,29]. Pre- and post-insertion morphostructural assessment performed with stereomicroscope and SEM showed no modification for all the miniscrews tested.

The aim of the present study was to evaluate the insertion torque values that are realized using orthodontic miniscrews in different bone thicknesses. Some authors report as reference values 5–10 Ncm, and over 10 Ncm can result in damage at the level of bone tissue [5,17,30,31].

A systematic review, however, has shown that there is no evidence to support this claim and to suggest there are more efficient insertion torques [8].

The authors of this article applied 40 miniscrews in four different substrates that simulated four different clinical conditions of human bone [29].

Previous authors analyzed the MIT values necessary to insert self-tapping and self-drilling screws in synthetic bone blocks. The values obtained by self-drilling for the bone block that represents the clinical condition (a thickness of 2 mm—40 PCF—range: 6.43 ± 2.09 Ncm) were similar to those obtained in our study (6.8 Ncm +/− 0.5) [5].

The results showed that the insertion torque values above 10 Ncm were realized only in the 40 PCF sample that represents cortical bone thickness: a rare clinical condition. The mean cortical thickness was recorded by Miyamoto et al. to be 1.49 +/− 0.34 mm for the maxilla and 2.22 +/− 0.47 mm for the mandible [24].

In the samples that represent the most frequent clinical conditions (30 PCF with 2 mm 50 PCF and 30 PCF with 4 mm 50 PCF), the torque values analyzed had values between 6.8 Ncm +/− 0.5 Ncm and 10.0 Ncm +/− 0.8. These values are like those reported in a recent study where the authors tested pullout strength of self-drilling miniscrews in samples with different cortical thicknesses. However, in the study the authors do not specific the means utilized to measure the insertion torque [29]. The insertion site with the optimal anatomical properties, according to some authors, is the vestibular bone corresponding to the distal root of the second molar, with a 4 mm vestibular screw inserted at the enamel–cement junction [2,10,32]. Considering the cortical bone thickness of optimal insertion sites, predrilling is always recommended to avoid a high-density insertion pair that could cause [33] heat development and consequent pathogenic noxa of the site [34,35,36]. Predrilling in all high-bone-density regions (with 3–4 mm bone cortical thickness), such as the jaw, the median parts of the upper alveolar crest and palate, is therefore required for the clinical application of miniscrews [13,37,38,39]. From morphostructural analysis with SEM and stereomicroscope, following the insertion and removal of the self-drilling miniscrew in bone substrates of different densities, no nicks or cuts were detected in any of the bone specimens. This result is of significance for specimens with 30 PCF density and with 50 PCF density cortical 4 mm thick, conditions in which, in vivo, the manufacturer recommends the insertion of self-drilling miniscrews with predrilled holes [5,32].

In samples with 20 PCF density, maximum torque values were reached below 1 mm deep and had a linear pattern, without dropping below 2 Ncm.

In samples with 40 PCF density, the torque had an incremental trend throughout the insertion thickness. The maximum torque value was above 19 Ncm.

These results lead us to conclude that in the samples of 20 PCF and 40 PCF, the detected torque value did not meet the standards required by the literature; the miniscrew inserted in such conditions, in vivo, would most likely fail, for lack of primary stability in the 20 PCF specimen, for development of pathogenic noxa in the 40 PCF specimen [34,35]. Both conditions represent a rare clinical condition.

**Figure 4:**
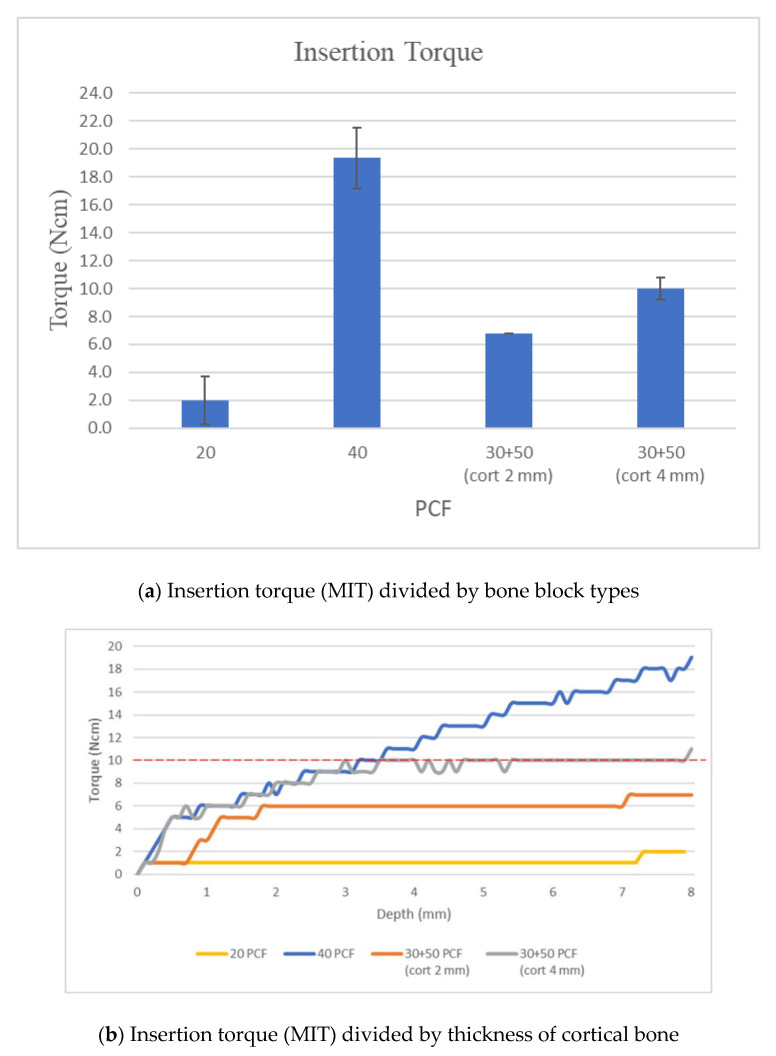
Graphs showing the results of the torque tester of screws on the artificial bone blocks. Maximum insertion torque depending on the thickness of cortical bone.

In samples with 30 PCF density and 2 mm cortical 50 PCF, torque values had an incremental trend until 2 mm of insertion, with increasing values of up to 6.8 +/− 0.5 Ncm, which remained constant until fully inserted (Figure 4).

In samples with 30 PCF density and 4 mm cortical 50 PCF, torque values had an incremental performance until 3.5 mm of insertion and retained the value of 10 +/− 0.8 Ncm until fully inserted. For these reasons, according to the present study, it is believed that the self-drilling mini-implants in steel can be connected according to the factory protocol in bone with cortex up to 4 mm without developing a torque value greater than 10 Ncm and therefore pathogenic noxa.

Based on in vitro study, the hypothesis was rejected, the self-drilling miniscrews have mechanical properties to enable drilling of the cortical bone without the generation of excessive torque and bone damage. Further studies in vivo are recommended to confirm these results.

## 6. Conclusions

Insertion torque values detected for bone specimens with 30 PCF density and bone cortical thickness at 50 PCF density of 2 mm and 4 mm are between 5 and 10 Ncm, which reduces the risk of failure of the miniscrews.

The morphostructural analysis, carried out with stereomicroscope and SEM, found no nicks or modifications of the miniscrew–substrate cutting edge in any of the bone samples at different densities, showing the appropriate mechanical properties of self-drilling miniscrews.

Through the in vitro study, the authors evaluated the ability of the screw to perforate the artificial blocks with different bone thicknesses that mimicked the density of the cortical bone represented in vivo. The advantages of the in vitro study are to reduce the clinical variables and to more accurately evaluate the mechanical capabilities of the miniscrews. However, this in vitro study has some limitations compared with clinical cases:

- Clinically, it is difficult to evaluate the thickness of the cortex and its density;
- The perpendicular insertion of the miniscrew is very difficult;
- The force applied to insert the miniscrew is operator-dependent.

Further in vitro studies are needed to evaluate the torque of the miniscrews during their insertion at different angles.

## Figures and Tables

**Table 1:** Mean values and standard deviations (SD) for insertion torque (MIT) divided by bone block types.

Insertion Torque				
PCF	20	40	30 + 50 (Cort 2 mm)	30 + 50 (Cort 4 mm)
mean	2.0	19.3	6.8	10.0
sd	0.8	1.5	0.5	0.8
